# (4′-Ferrocenyl-2,2′:6′,2′′-terpyridine-κ^3^ *N*,*N*′,*N*′′)(1,10-phenanthroline-κ^2^ *N*,*N*′)copper(II) bis(perchlorate) acetonitrile solvate

**DOI:** 10.1107/S1600536809020753

**Published:** 2009-06-06

**Authors:** Si-Ping Tang, Dai-Zhi Kuang, Yong-Lan Feng, Man-Sheng Chen, Wei Li

**Affiliations:** a Key Laboratory of Functional Organometallic Materials, Hengyang Normal University, Hengyang, Hunan 421008, People’s Republic of China.

## Abstract

The title complex, [CuFe(C_5_H_5_)(C_20_H_14_N_3_)(C_12_H_8_N_2_)](ClO_4_)_2_·C_2_H_3_N, consists of a mononuclear [Cu(C_12_H_8_N_2_)(C_25_H_19_FeN_3_)]^2+^ cation, two ClO_4_^−^ anions (one of which is disordered over two positions with equal occupancy) and one CH_3_CN solvent mol­ecule. The Cu^II^ center has a distorted square-pyramidal coordination with three N atoms of the 4′-ferrocenyl-2,2′:6′,2′′- terpyridine (fctpy) ligand and one 1,10-phenanthroline (phen) N atom in the basal plane and a second phen N atom in the apical position with an axial distance of 2.254 (4) Å. The disordered ClO_4_^−^ anion is weakly coordin­ated to the Cu^II^ ion with a Cu—O distance of 2.766 (11) Å. The two cyclo­penta­dienyl rings of the ferrocenyl group are almost eclipsed with a deviation of 4.7 (1) °, and are involved in inter­molecular π–π inter­actions with the outer pyridyl rings of the fctpy ligands [centroid–centroid distance = 3.759 (2) Å.].

## Related literature

For related complexes of the fctpy ligand, see: Aguado *et al.* (2005 ▶); Constable *et al.* (1994 ▶); Farlow *et al.* (1993 ▶); Tang & Kuang (2007 ▶).
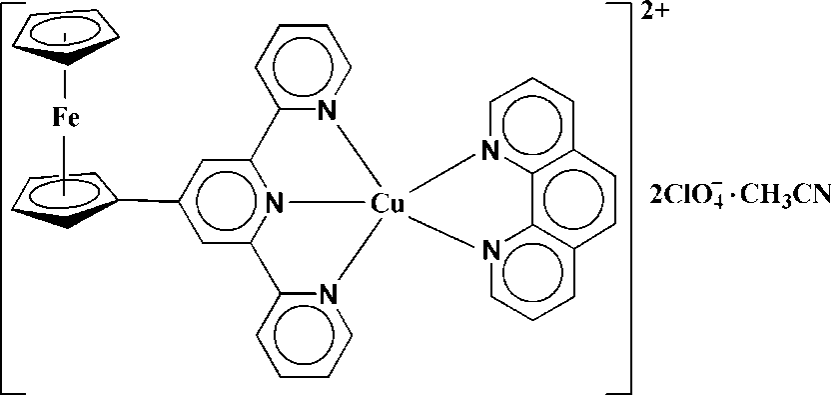


## Experimental

### 

#### Crystal data

[CuFe(C_5_H_5_)(C_20_H_14_N_3_)(C_12_H_8_N_2_)](ClO_4_)_2_·C_2_H_3_N
*M* *_r_* = 900.98
Monoclinic, 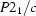

*a* = 13.5554 (9) Å
*b* = 12.1087 (8) Å
*c* = 23.1754 (14) Å
β = 97.577 (1)°
*V* = 3770.8 (4) Å^3^
*Z* = 4
Mo *K*α radiation
μ = 1.15 mm^−1^
*T* = 295 K
0.24 × 0.18 × 0.07 mm

#### Data collection

Bruker SMART APEX area-detector diffractometer
Absorption correction: multi-scan (*SADABS*; Sheldrick, 1996 ▶) *T*_min_ = 0.769, *T*_max_ = 0.924
19185 measured reflections
7358 independent reflections
4622 reflections with *I* > 2σ(*I*)
*R*_int_ = 0.039

#### Refinement

*R*[*F* ^2^ > 2σ(*F* ^2^)] = 0.064
*wR*(*F* ^2^) = 0.189
*S* = 1.03
7358 reflections
552 parameters
74 restraints
H-atom parameters constrained
Δρ_max_ = 0.85 e Å^−3^
Δρ_min_ = −0.43 e Å^−3^

### 

Data collection: *SMART* (Bruker, 2002 ▶); cell refinement: *SAINT* (Bruker, 2002 ▶); data reduction: *SAINT*; program(s) used to solve structure: *SHELXS97* (Sheldrick, 2008 ▶); program(s) used to refine structure: *SHELXL97* (Sheldrick, 2008 ▶); molecular graphics: *SHELXTL* (Sheldrick, 2008 ▶); software used to prepare material for publication: *SHELXTL*.

## Supplementary Material

Crystal structure: contains datablocks I, global. DOI: 10.1107/S1600536809020753/at2803sup1.cif

Structure factors: contains datablocks I. DOI: 10.1107/S1600536809020753/at2803Isup2.hkl

Additional supplementary materials:  crystallographic information; 3D view; checkCIF report

## Figures and Tables

**Table 1:** Selected bond lengths (Å)

Cu1—N2	1.930 (3)
Cu1—N4	2.000 (4)
Cu1—N1	2.051 (4)
Cu1—N3	2.064 (4)

**Fig. 1.**
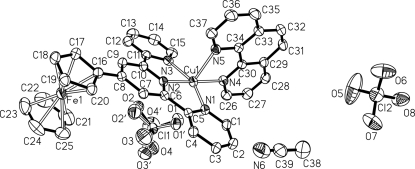
The title molecule with displacement ellipsoids drawn at the 30% probability level.The H atoms and the minor component of the disorder has been omitted for clarity.

## supplementary crystallographic information

### Comment

4'-Ferrocenyl-2,2':6',2''-terpyridine (fctpy) have rencently been paid more attentions because of its coordinating abilities towards transition metal ions, such as Au^I^, Ru^II^, Co^II^, Fe^II^ and Cu^II^ metals (Aguado *et al.*, 2005; Constable *et al.*, 1994; Farlow *et al.*, 1993, Tang & Kuang, 2007), and some of its complexes exhibited interesting electrochemical properties. Ongoing our work at the study of fctpy complexes (Tang & Kuang, 2007), a new Cu^II^ complex of incorporating fctpy and 1,10-phenanthroline (phen) as mixed-ligands is here reported.

As shown in Fig. 1, in the title complex, the Cu ^II^ center is five- coordinated by three N atoms from the fctpy ligand and two N atoms from the phen ligand to give a distorted square pyramidal geometry. The N5 atom from the phen ligand is located at the apical position and form the Cu—N distance of 2.254 (4) Å, which is markedly longer than values in the basal plane [1.930 (3)—2.064 (4) Å] resulting from the Jahn-Teller effect of divalent copper ion. The O1 atom of one ClO_4_^-^ anion is werkly coordinated to the Cu^II^ center with a distance of 2.766 (11) Å. The central pyridine of the fctpy ligand are almost coplanar with the two outer pyridines [dihedral angles of 6.2 (1) ° and 6.8 (1) °, respectively], however, which form a larger tilt angle of 9.3 (2) ° from its linking cyclopentadienyl ring. The two cyclopentadienyl rings of the ferrocenyl group is almost eclipsed with a deviation of 4.7 (1) °.

In the crystal packing, the cyclopentadienyl rings attached to the central pyridines and the adjacent outer N1-pyridyl rings of the fctpy ligands form intermolecular π···π interactions with the centroid-to-centroid distances of 3.759 (2) Å.

### Experimental

A solution of copper perchlorates hexahydrate (18.5 mg, 0.05 mmol), fctpy (21.0 mg, 0.05 mmol) and 1,10-phenanthroline (9.9 mg, 0.05 mmol) in acetonitrile (7 ml) was stirred for 4 h. The resulting solution was filtered off and the filtrate was diffused by diethylether evaporation to give dark-purple sheet crystals of the title complex after three days [yield: 25 mg (55%)].

### Refinement

One of the perchlorate anions is two-fold disordered and the site-occupancy factors of its O atoms (constrained to sum to unity) were refined to 0.503 (10):0.497 (10). The carbon-bound H atoms were placed at calculated positions (C—H = 0.93 Å) and refined as riding, with *U*(H) = 1.2*U*_eq_(C) for phenyl and cyclopentadienyl H atoms, and C—H = 0.96 Å and *U*_iso_ = 1.5*U*_eq_ (C) for methyl H atoms.

### Crystal data

**Table:**

[CuFe(C_5_H_5_)(C_20_H_14_N_3_)(C_12_H_8_N_2_)](ClO_4_)_2_·C_2_H_3_N	*F*(000) = 1836
*M**_r_* = 900.98	*D*_x_ = 1.587 Mg m^−^^3^
Monoclinic, *P*2_1_/*c*	Mo *K*α radiation, λ = 0.71073 Å
Hall symbol: -P 2ybc	Cell parameters from 3235 reflections
*a* = 13.5554 (9) Å	θ = 2.3–22.3°
*b* = 12.1087 (8) Å	µ = 1.15 mm^−^^1^
*c* = 23.1754 (14) Å	*T* = 295 K
β = 97.577 (1)°	Sheet, dark-purple
*V* = 3770.8 (4) Å^3^	0.24 × 0.18 × 0.07 mm
*Z* = 4	

### Data collection

**Table:**

Bruker SMART APEX area-detector diffractometer	7358 independent reflections
Radiation source: fine-focus sealed tube	4622 reflections with *I* > 2σ(*I*)
graphite	*R*_int_ = 0.039
φ and ω scans	θ_max_ = 26.0°, θ_min_ = 1.9°
Absorption correction: multi-scan (*SADABS*; Sheldrick, 1996)	*h* = −16→16
*T*_min_ = 0.769, *T*_max_ = 0.924	*k* = −9→14
19185 measured reflections	*l* = −28→27

### Refinement

**Table:**

Refinement on *F*^2^	Primary atom site location: structure-invariant direct methods
Least-squares matrix: full	Secondary atom site location: difference Fourier map
*R*[*F*^2^ > 2σ(*F*^2^)] = 0.064	Hydrogen site location: inferred from neighbouring sites
*wR*(*F*^2^) = 0.189	H-atom parameters constrained
*S* = 1.03	*w* = 1/[σ^2^(*F*_o_^2^) + (0.1061*P*)^2^] where *P* = (*F*_o_^2^ + 2*F*_c_^2^)/3
7358 reflections	(Δ/σ)_max_ < 0.001
552 parameters	Δρ_max_ = 0.85 e Å^−^^3^
74 restraints	Δρ_min_ = −0.43 e Å^−^^3^

### Special details

**Table:**

Geometry. All esds (except the esd in the dihedral angle between two l.s. planes) are estimated using the full covariance matrix. The cell esds are taken into account individually in the estimation of esds in distances, angles and torsion angles; correlations between esds in cell parameters are only used when they are defined by crystal symmetry. An approximate (isotropic) treatment of cell esds is used for estimating esds involving l.s. planes.
Refinement. Refinement of F^2^ against ALL reflections. The weighted R-factor wR and goodness of fit S are based on F^2^, conventional R-factors R are based on F, with F set to zero for negative F^2^. The threshold expression of F^2^ > 2sigma(F^2^) is used only for calculating R-factors(gt) etc. and is not relevant to the choice of reflections for refinement. R-factors based on F^2^ are statistically about twice as large as those based on F, and R- factors based on ALL data will be even larger.

### Fractional atomic coordinates and isotropic or equivalent isotropic displacement parameters (Å^2^)

**Table:**

	*x*	*y*	*z*	*U*_iso_*/*U*_eq_	Occ. (<1)
Fe1	0.56440 (6)	0.56049 (6)	0.30991 (3)	0.0640 (3)	
Cu1	0.79531 (4)	0.19291 (4)	0.55087 (2)	0.0531 (2)	
N1	0.6492 (3)	0.1506 (3)	0.55239 (15)	0.0524 (9)	
N2	0.7317 (3)	0.3010 (3)	0.49711 (15)	0.0495 (9)	
N3	0.9173 (3)	0.2550 (3)	0.51690 (16)	0.0562 (10)	
N4	0.8606 (3)	0.0866 (3)	0.61003 (16)	0.0536 (9)	
N5	0.8048 (3)	0.2962 (3)	0.63236 (16)	0.0556 (10)	
C1	0.6120 (4)	0.0650 (4)	0.5793 (2)	0.0597 (12)	
H1	0.6561	0.0165	0.6004	0.072*	
C2	0.5128 (4)	0.0462 (4)	0.5770 (2)	0.0649 (13)	
H2	0.4898	−0.0136	0.5966	0.078*	
C3	0.4465 (4)	0.1170 (4)	0.5453 (2)	0.0646 (13)	
H3	0.3783	0.1052	0.5428	0.078*	
C4	0.4832 (4)	0.2052 (4)	0.5174 (2)	0.0590 (12)	
H4	0.4400	0.2547	0.4963	0.071*	
C5	0.5846 (3)	0.2193 (3)	0.52107 (17)	0.0468 (10)	
C6	0.6325 (3)	0.3096 (3)	0.49141 (18)	0.0488 (10)	
C7	0.5843 (3)	0.3947 (4)	0.45853 (19)	0.0535 (11)	
H7	0.5153	0.4003	0.4543	0.064*	
C8	0.6406 (4)	0.4715 (3)	0.43205 (18)	0.0528 (11)	
C9	0.7441 (4)	0.4569 (4)	0.43842 (19)	0.0560 (12)	
H9	0.7834	0.5058	0.4206	0.067*	
C10	0.7882 (3)	0.3701 (4)	0.47106 (18)	0.0496 (10)	
C11	0.8954 (3)	0.3417 (4)	0.48049 (19)	0.0533 (11)	
C34	0.8403 (3)	0.2321 (4)	0.67855 (19)	0.0504 (11)	
C12	0.9674 (4)	0.3933 (4)	0.4545 (2)	0.0660 (13)	
H12	0.9514	0.4533	0.4300	0.079*	
C13	1.0637 (4)	0.3555 (5)	0.4648 (2)	0.0731 (15)	
H13	1.1133	0.3897	0.4472	0.088*	
C14	1.0864 (4)	0.2673 (5)	0.5012 (3)	0.0746 (15)	
H14	1.1512	0.2407	0.5084	0.090*	
C15	1.0110 (4)	0.2186 (4)	0.5272 (2)	0.0643 (13)	
H15	1.0259	0.1593	0.5523	0.077*	
C16	0.5928 (4)	0.5633 (4)	0.3980 (2)	0.0560 (12)	
C17	0.6429 (4)	0.6521 (4)	0.3735 (2)	0.0637 (13)	
H17	0.7113	0.6639	0.3781	0.076*	
C18	0.5702 (4)	0.7185 (4)	0.3411 (2)	0.0701 (14)	
H18	0.5825	0.7820	0.3207	0.084*	
C19	0.4771 (4)	0.6732 (4)	0.3449 (2)	0.0670 (14)	
H19	0.4164	0.7011	0.3275	0.080*	
C20	0.4907 (4)	0.5772 (4)	0.3798 (2)	0.0606 (12)	
H20	0.4404	0.5309	0.3891	0.073*	
C21	0.6119 (11)	0.4136 (8)	0.2807 (4)	0.132 (4)	
H21	0.6468	0.3590	0.3030	0.159*	
C22	0.6541 (7)	0.5022 (11)	0.2534 (5)	0.127 (3)	
H22	0.7253	0.5174	0.2545	0.152*	
C23	0.5772 (10)	0.5624 (7)	0.2238 (3)	0.119 (3)	
H23	0.5836	0.6254	0.2017	0.143*	
C24	0.4883 (6)	0.5122 (10)	0.2330 (3)	0.116 (3)	
H24	0.4248	0.5353	0.2177	0.139*	
C25	0.5113 (10)	0.4214 (8)	0.2689 (4)	0.123 (3)	
H25	0.4656	0.3740	0.2826	0.148*	
C26	0.8881 (4)	−0.0146 (4)	0.5985 (2)	0.0657 (13)	
H26	0.8796	−0.0391	0.5601	0.079*	
C27	0.9297 (4)	−0.0862 (4)	0.6426 (3)	0.0705 (15)	
H27	0.9495	−0.1568	0.6334	0.085*	
C28	0.9409 (4)	−0.0520 (4)	0.6987 (2)	0.0666 (14)	
H28	0.9675	−0.0999	0.7281	0.080*	
C29	0.9130 (4)	0.0542 (4)	0.7128 (2)	0.0586 (12)	
C30	0.8712 (3)	0.1215 (4)	0.66610 (19)	0.0513 (11)	
C31	0.9213 (4)	0.0969 (5)	0.7707 (2)	0.0726 (15)	
H31	0.9503	0.0535	0.8015	0.087*	
C32	0.8885 (4)	0.1977 (5)	0.7818 (2)	0.0738 (15)	
H32	0.8934	0.2222	0.8201	0.089*	
C33	0.8464 (4)	0.2678 (4)	0.7364 (2)	0.0611 (12)	
C35	0.8131 (4)	0.3765 (5)	0.7455 (2)	0.0759 (15)	
H35	0.8154	0.4043	0.7830	0.091*	
C36	0.7776 (5)	0.4401 (4)	0.6987 (3)	0.0792 (16)	
H36	0.7542	0.5111	0.7041	0.095*	
C37	0.7767 (4)	0.3976 (4)	0.6424 (2)	0.0649 (13)	
H37	0.7554	0.4430	0.6109	0.078*	
N6	0.0763 (5)	0.3621 (5)	0.6460 (3)	0.113 (2)	
Cl2	0.29184 (12)	0.20991 (13)	0.88974 (7)	0.0857 (5)	
O5	0.3650 (7)	0.2111 (8)	0.8565 (4)	0.246 (5)	
O6	0.2824 (4)	0.3091 (4)	0.9187 (3)	0.141 (2)	
O7	0.1973 (4)	0.1838 (4)	0.8571 (2)	0.1275 (19)	
O8	0.3153 (4)	0.1247 (3)	0.9304 (2)	0.1123 (16)	
C38	0.1646 (7)	0.2120 (7)	0.7127 (3)	0.122 (3)	
H38A	0.2339	0.2306	0.7210	0.183*	
H38B	0.1578	0.1414	0.6938	0.183*	
H38C	0.1360	0.2088	0.7484	0.183*	
C39	0.1138 (6)	0.2951 (6)	0.6751 (3)	0.0882 (19)	
Cl1	0.79250 (11)	0.04474 (12)	0.41104 (6)	0.0730 (4)	
O1	0.8078 (9)	0.0235 (9)	0.4721 (3)	0.092 (4)	0.497 (10)
O2	0.8610 (8)	0.1286 (8)	0.3996 (4)	0.104 (4)	0.497 (10)
O3	0.6987 (7)	0.0976 (10)	0.3933 (5)	0.149 (5)	0.497 (10)
O4	0.7975 (11)	−0.0533 (8)	0.3809 (5)	0.150 (5)	0.497 (10)
O1'	0.7641 (8)	0.0434 (9)	0.4675 (3)	0.078 (3)	0.503 (10)
O4'	0.8813 (8)	−0.0103 (11)	0.4016 (6)	0.173 (6)	0.503 (10)
O2'	0.8038 (10)	0.1583 (6)	0.3941 (5)	0.119 (4)	0.503 (10)
O3'	0.7185 (7)	−0.0064 (8)	0.3701 (4)	0.108 (4)	0.503 (10)

### Atomic displacement parameters (Å^2^)

**Table:**

	*U*^11^	*U*^22^	*U*^33^	*U*^12^	*U*^13^	*U*^23^
Fe1	0.0775 (5)	0.0658 (5)	0.0484 (4)	0.0035 (4)	0.0073 (4)	0.0001 (3)
Cu1	0.0563 (4)	0.0574 (4)	0.0447 (3)	0.0038 (3)	0.0040 (3)	0.0058 (2)
N1	0.062 (2)	0.054 (2)	0.041 (2)	−0.0043 (18)	0.0081 (18)	0.0020 (17)
N2	0.056 (2)	0.053 (2)	0.0395 (19)	−0.0003 (17)	0.0072 (17)	0.0025 (16)
N3	0.053 (2)	0.065 (2)	0.051 (2)	−0.0010 (19)	0.0058 (18)	−0.0063 (19)
N4	0.053 (2)	0.056 (2)	0.052 (2)	0.0048 (18)	0.0066 (18)	0.0005 (18)
N5	0.060 (2)	0.056 (2)	0.050 (2)	0.0045 (18)	0.0024 (18)	0.0033 (18)
C1	0.078 (4)	0.052 (3)	0.050 (3)	0.001 (2)	0.011 (2)	0.010 (2)
C2	0.074 (4)	0.064 (3)	0.059 (3)	−0.015 (3)	0.018 (3)	0.007 (2)
C3	0.064 (3)	0.077 (3)	0.055 (3)	−0.017 (3)	0.015 (2)	0.007 (3)
C4	0.062 (3)	0.065 (3)	0.051 (3)	−0.001 (2)	0.008 (2)	0.008 (2)
C5	0.054 (3)	0.051 (2)	0.036 (2)	−0.002 (2)	0.0043 (19)	0.0011 (18)
C6	0.054 (3)	0.052 (3)	0.040 (2)	−0.003 (2)	0.0040 (19)	−0.0005 (19)
C7	0.053 (3)	0.056 (3)	0.050 (3)	−0.002 (2)	0.003 (2)	0.007 (2)
C8	0.067 (3)	0.047 (2)	0.042 (2)	−0.004 (2)	0.003 (2)	0.002 (2)
C9	0.069 (3)	0.052 (3)	0.048 (3)	−0.007 (2)	0.010 (2)	0.005 (2)
C10	0.052 (3)	0.056 (3)	0.041 (2)	−0.008 (2)	0.009 (2)	−0.002 (2)
C11	0.057 (3)	0.054 (3)	0.049 (3)	−0.005 (2)	0.008 (2)	−0.008 (2)
C34	0.053 (3)	0.049 (2)	0.049 (3)	−0.004 (2)	0.006 (2)	0.003 (2)
C12	0.072 (4)	0.064 (3)	0.065 (3)	−0.008 (3)	0.018 (3)	−0.007 (3)
C13	0.056 (3)	0.087 (4)	0.081 (4)	−0.011 (3)	0.027 (3)	−0.019 (3)
C14	0.055 (3)	0.084 (4)	0.085 (4)	0.011 (3)	0.011 (3)	−0.016 (3)
C15	0.057 (3)	0.071 (3)	0.064 (3)	0.005 (3)	0.007 (3)	−0.014 (3)
C16	0.068 (3)	0.051 (3)	0.049 (3)	−0.001 (2)	0.008 (2)	0.001 (2)
C17	0.072 (3)	0.059 (3)	0.060 (3)	−0.007 (3)	0.008 (3)	0.011 (2)
C18	0.091 (4)	0.055 (3)	0.063 (3)	0.004 (3)	0.004 (3)	0.013 (2)
C19	0.078 (4)	0.062 (3)	0.060 (3)	0.013 (3)	0.004 (3)	0.005 (2)
C20	0.072 (3)	0.055 (3)	0.055 (3)	0.006 (2)	0.009 (2)	0.003 (2)
C21	0.186 (11)	0.109 (7)	0.097 (6)	0.053 (7)	0.001 (7)	−0.034 (5)
C22	0.111 (6)	0.159 (9)	0.119 (7)	−0.005 (7)	0.044 (6)	−0.057 (7)
C23	0.188 (10)	0.119 (6)	0.056 (4)	0.012 (7)	0.036 (5)	0.001 (4)
C24	0.107 (6)	0.173 (9)	0.062 (4)	0.017 (6)	−0.013 (4)	−0.036 (5)
C25	0.181 (10)	0.108 (6)	0.084 (6)	−0.046 (6)	0.029 (6)	−0.039 (5)
C26	0.070 (3)	0.063 (3)	0.064 (3)	0.007 (3)	0.007 (3)	−0.006 (3)
C27	0.070 (3)	0.058 (3)	0.083 (4)	0.014 (3)	0.008 (3)	0.009 (3)
C28	0.059 (3)	0.070 (3)	0.069 (4)	0.005 (3)	0.003 (3)	0.019 (3)
C29	0.061 (3)	0.062 (3)	0.052 (3)	−0.001 (2)	0.002 (2)	0.011 (2)
C30	0.050 (3)	0.053 (3)	0.050 (3)	−0.003 (2)	0.004 (2)	0.002 (2)
C31	0.081 (4)	0.084 (4)	0.050 (3)	−0.001 (3)	−0.003 (3)	0.016 (3)
C32	0.088 (4)	0.087 (4)	0.045 (3)	−0.011 (3)	0.007 (3)	−0.003 (3)
C33	0.064 (3)	0.067 (3)	0.051 (3)	−0.007 (2)	0.005 (2)	−0.001 (2)
C35	0.092 (4)	0.075 (4)	0.061 (3)	0.002 (3)	0.011 (3)	−0.020 (3)
C36	0.098 (4)	0.060 (3)	0.079 (4)	0.006 (3)	0.010 (3)	−0.016 (3)
C37	0.072 (3)	0.056 (3)	0.065 (3)	0.005 (3)	0.004 (3)	−0.002 (2)
N6	0.138 (6)	0.103 (5)	0.095 (5)	−0.013 (4)	0.006 (4)	−0.019 (4)
Cl2	0.0875 (11)	0.0783 (10)	0.0941 (11)	0.0097 (8)	0.0218 (9)	−0.0006 (8)
O5	0.219 (8)	0.275 (10)	0.285 (9)	0.055 (7)	0.189 (8)	0.112 (8)
O6	0.116 (4)	0.085 (3)	0.208 (6)	0.023 (3)	−0.037 (4)	−0.043 (3)
O7	0.140 (4)	0.136 (4)	0.094 (3)	0.017 (3)	−0.032 (3)	−0.027 (3)
O8	0.123 (4)	0.075 (3)	0.129 (4)	−0.014 (3)	−0.020 (3)	0.012 (3)
C38	0.140 (7)	0.124 (6)	0.105 (6)	−0.013 (5)	0.028 (5)	0.007 (5)
C39	0.107 (5)	0.094 (5)	0.067 (4)	−0.019 (4)	0.026 (4)	−0.026 (4)
Cl1	0.0828 (9)	0.0817 (9)	0.0562 (7)	−0.0047 (7)	0.0158 (7)	−0.0018 (6)
O1	0.098 (5)	0.100 (5)	0.075 (5)	0.012 (4)	0.003 (4)	0.012 (4)
O2	0.094 (5)	0.122 (6)	0.099 (5)	−0.022 (4)	0.030 (4)	0.003 (4)
O3	0.138 (7)	0.161 (7)	0.146 (7)	0.012 (5)	0.007 (4)	0.001 (5)
O4	0.170 (7)	0.140 (6)	0.140 (6)	0.009 (5)	0.018 (5)	−0.031 (4)
O1'	0.087 (5)	0.090 (5)	0.062 (4)	−0.011 (4)	0.025 (3)	0.001 (3)
O4'	0.155 (7)	0.181 (7)	0.187 (7)	0.018 (5)	0.036 (5)	0.003 (5)
O2'	0.140 (6)	0.111 (5)	0.107 (5)	0.005 (4)	0.021 (4)	0.013 (4)
O3'	0.109 (5)	0.124 (6)	0.087 (5)	−0.018 (4)	−0.001 (4)	−0.015 (4)

### Geometric parameters (Å, °)

**Table:**

Fe1—C25	2.020 (7)	C16—C17	1.429 (6)
Fe1—C20	2.021 (5)	C17—C18	1.409 (7)
Fe1—C23	2.025 (7)	C17—H17	0.9300
Fe1—C24	2.025 (6)	C18—C19	1.390 (8)
Fe1—C16	2.028 (5)	C18—H18	0.9300
Fe1—C22	2.028 (8)	C19—C20	1.414 (6)
Fe1—C17	2.030 (5)	C19—H19	0.9300
Fe1—C21	2.038 (8)	C20—H20	0.9300
Fe1—C18	2.043 (5)	C21—C25	1.358 (12)
Fe1—C19	2.043 (5)	C21—C22	1.405 (13)
Cu1—N2	1.930 (3)	C21—H21	0.9300
Cu1—N4	2.000 (4)	C22—C23	1.378 (12)
Cu1—N1	2.051 (4)	C22—H22	0.9800
Cu1—N3	2.064 (4)	C23—C24	1.390 (11)
Cu1—N5	2.254 (4)	C23—H23	0.9300
Cu1—O1	2.766 (11)	C24—C25	1.390 (11)
N1—C1	1.341 (6)	C24—H24	0.9300
N1—C5	1.348 (5)	C25—H25	0.9300
N2—C10	1.331 (5)	C26—C27	1.400 (7)
N2—C6	1.339 (6)	C26—H26	0.9300
N3—C15	1.336 (6)	C27—C28	1.354 (7)
N3—C11	1.355 (6)	C27—H27	0.9300
N4—C26	1.319 (6)	C28—C29	1.392 (7)
N4—C30	1.356 (6)	C28—H28	0.9300
N5—C37	1.316 (6)	C29—C30	1.412 (6)
N5—C34	1.358 (5)	C29—C31	1.430 (7)
C1—C2	1.359 (7)	C31—C32	1.335 (7)
C1—H1	0.9300	C31—H31	0.9300
C2—C3	1.382 (7)	C32—C33	1.412 (7)
C2—H2	0.9300	C32—H32	0.9300
C3—C4	1.375 (6)	C33—C35	1.417 (7)
C3—H3	0.9300	C35—C36	1.365 (7)
C4—C5	1.377 (6)	C35—H35	0.9300
C4—H4	0.9300	C36—C37	1.400 (7)
C5—C6	1.485 (6)	C36—H36	0.9300
C6—C7	1.392 (6)	C37—H37	0.9300
C7—C8	1.396 (6)	N6—C39	1.131 (8)
C7—H7	0.9300	Cl2—O5	1.333 (7)
C8—C9	1.403 (7)	Cl2—O6	1.390 (5)
C8—C16	1.464 (6)	Cl2—O8	1.404 (4)
C9—C10	1.384 (6)	Cl2—O7	1.435 (5)
C9—H9	0.9300	C38—C39	1.447 (10)
C10—C11	1.480 (6)	C38—H38A	0.9600
C11—C12	1.364 (7)	C38—H38B	0.9600
C34—C33	1.401 (6)	C38—H38C	0.9600
C34—C30	1.443 (6)	Cl1—O4	1.384 (7)
C12—C13	1.375 (7)	Cl1—O1'	1.413 (6)
C12—H12	0.9300	Cl1—O4'	1.418 (8)
C13—C14	1.370 (8)	Cl1—O2	1.424 (7)
C13—H13	0.9300	Cl1—O1	1.427 (7)
C14—C15	1.384 (8)	Cl1—O3'	1.429 (6)
C14—H14	0.9300	Cl1—O3	1.434 (7)
C15—H15	0.9300	Cl1—O2'	1.443 (7)
C16—C20	1.403 (7)		
			
C25—Fe1—C20	106.1 (3)	C8—C16—Fe1	122.8 (3)
C25—Fe1—C23	67.5 (3)	C18—C17—C16	107.7 (5)
C20—Fe1—C23	154.6 (4)	C18—C17—Fe1	70.2 (3)
C25—Fe1—C24	40.2 (3)	C16—C17—Fe1	69.3 (3)
C20—Fe1—C24	119.1 (3)	C18—C17—H17	126.1
C23—Fe1—C24	40.1 (3)	C16—C17—H17	126.1
C25—Fe1—C16	119.8 (3)	Fe1—C17—H17	125.9
C20—Fe1—C16	40.53 (19)	C19—C18—C17	108.6 (4)
C23—Fe1—C16	164.2 (4)	C19—C18—Fe1	70.1 (3)
C24—Fe1—C16	154.0 (4)	C17—C18—Fe1	69.3 (3)
C25—Fe1—C22	67.2 (4)	C19—C18—H18	125.7
C20—Fe1—C22	162.5 (5)	C17—C18—H18	125.7
C23—Fe1—C22	39.8 (3)	Fe1—C18—H18	126.5
C24—Fe1—C22	67.0 (3)	C18—C19—C20	108.0 (5)
C16—Fe1—C22	127.3 (4)	C18—C19—Fe1	70.1 (3)
C25—Fe1—C17	156.6 (4)	C20—C19—Fe1	68.8 (3)
C20—Fe1—C17	68.3 (2)	C18—C19—H19	126.0
C23—Fe1—C17	127.2 (4)	C20—C19—H19	126.0
C24—Fe1—C17	162.6 (4)	Fe1—C19—H19	126.6
C16—Fe1—C17	41.22 (18)	C16—C20—C19	108.7 (5)
C22—Fe1—C17	110.9 (3)	C16—C20—Fe1	70.0 (3)
C25—Fe1—C21	39.1 (3)	C19—C20—Fe1	70.5 (3)
C20—Fe1—C21	124.4 (4)	C16—C20—H20	125.7
C23—Fe1—C21	67.1 (4)	C19—C20—H20	125.7
C24—Fe1—C21	66.5 (3)	Fe1—C20—H20	125.5
C16—Fe1—C21	108.9 (3)	C25—C21—C22	108.4 (9)
C22—Fe1—C21	40.4 (4)	C25—C21—Fe1	69.7 (5)
C17—Fe1—C21	124.1 (4)	C22—C21—Fe1	69.4 (5)
C25—Fe1—C18	160.5 (4)	C25—C21—H21	125.8
C20—Fe1—C18	67.8 (2)	C22—C21—H21	125.8
C23—Fe1—C18	109.5 (3)	Fe1—C21—H21	126.6
C24—Fe1—C18	125.0 (3)	C23—C22—C21	107.5 (9)
C16—Fe1—C18	68.54 (19)	C23—C22—Fe1	70.0 (5)
C22—Fe1—C18	123.8 (4)	C21—C22—Fe1	70.2 (5)
C17—Fe1—C18	40.48 (19)	C23—C22—H22	126.2
C21—Fe1—C18	159.4 (5)	C21—C22—H22	126.2
C25—Fe1—C19	123.7 (4)	Fe1—C22—H22	126.2
C20—Fe1—C19	40.72 (19)	C22—C23—C24	107.9 (8)
C23—Fe1—C19	120.8 (3)	C22—C23—Fe1	70.2 (4)
C24—Fe1—C19	106.7 (3)	C24—C23—Fe1	69.9 (4)
C16—Fe1—C19	68.42 (19)	C22—C23—H23	126.0
C22—Fe1—C19	156.5 (5)	C24—C23—H23	126.0
C17—Fe1—C19	67.8 (2)	Fe1—C23—H23	125.4
C21—Fe1—C19	160.1 (5)	C25—C24—C23	107.9 (8)
C18—Fe1—C19	39.8 (2)	C25—C24—Fe1	69.7 (4)
N2—Cu1—N4	176.93 (15)	C23—C24—Fe1	69.9 (4)
N2—Cu1—N1	80.23 (15)	C25—C24—H24	126.1
N4—Cu1—N1	99.71 (15)	C23—C24—H24	126.1
N2—Cu1—N3	79.55 (16)	Fe1—C24—H24	125.9
N4—Cu1—N3	101.06 (16)	C21—C25—C24	108.4 (9)
N1—Cu1—N3	157.13 (14)	C21—C25—Fe1	71.2 (5)
N2—Cu1—N5	97.81 (14)	C24—C25—Fe1	70.1 (4)
N4—Cu1—N5	79.12 (14)	C21—C25—H25	125.8
N1—Cu1—N5	94.17 (14)	C24—C25—H25	125.8
N3—Cu1—N5	99.06 (14)	Fe1—C25—H25	124.5
N2—Cu1—O1	97.9 (2)	N4—C26—C27	121.6 (5)
N4—Cu1—O1	85.1 (2)	N4—C26—H26	119.2
N1—Cu1—O1	88.2 (3)	C27—C26—H26	119.2
N3—Cu1—O1	84.1 (3)	C28—C27—C26	119.7 (5)
N5—Cu1—O1	164.3 (2)	C28—C27—H27	120.1
C1—N1—C5	118.0 (4)	C26—C27—H27	120.1
C1—N1—Cu1	128.6 (3)	C27—C28—C29	120.5 (5)
C5—N1—Cu1	113.4 (3)	C27—C28—H28	119.8
C10—N2—C6	122.5 (4)	C29—C28—H28	119.8
C10—N2—Cu1	119.0 (3)	C28—C29—C30	116.6 (4)
C6—N2—Cu1	118.3 (3)	C28—C29—C31	124.3 (5)
C15—N3—C11	119.4 (4)	C30—C29—C31	119.0 (5)
C15—N3—Cu1	127.1 (4)	N4—C30—C29	122.4 (4)
C11—N3—Cu1	113.5 (3)	N4—C30—C34	118.9 (4)
C26—N4—C30	119.1 (4)	C29—C30—C34	118.7 (4)
C26—N4—Cu1	124.8 (3)	C32—C31—C29	121.6 (5)
C30—N4—Cu1	116.0 (3)	C32—C31—H31	119.2
C37—N5—C34	118.3 (4)	C29—C31—H31	119.2
C37—N5—Cu1	132.9 (3)	C31—C32—C33	121.2 (5)
C34—N5—Cu1	108.6 (3)	C31—C32—H32	119.4
N1—C1—C2	122.9 (5)	C33—C32—H32	119.4
N1—C1—H1	118.6	C34—C33—C32	119.7 (5)
C2—C1—H1	118.6	C34—C33—C35	116.7 (4)
C1—C2—C3	119.1 (4)	C32—C33—C35	123.6 (5)
C1—C2—H2	120.4	C36—C35—C33	119.5 (5)
C3—C2—H2	120.4	C36—C35—H35	120.3
C4—C3—C2	118.8 (5)	C33—C35—H35	120.3
C4—C3—H3	120.6	C35—C36—C37	119.5 (5)
C2—C3—H3	120.6	C35—C36—H36	120.3
C3—C4—C5	119.2 (5)	C37—C36—H36	120.3
C3—C4—H4	120.4	N5—C37—C36	122.7 (5)
C5—C4—H4	120.4	N5—C37—H37	118.6
N1—C5—C4	121.9 (4)	C36—C37—H37	118.6
N1—C5—C6	114.3 (4)	O5—Cl2—O6	113.1 (6)
C4—C5—C6	123.8 (4)	O5—Cl2—O8	106.0 (4)
N2—C6—C7	120.3 (4)	O6—Cl2—O8	109.7 (3)
N2—C6—C5	113.1 (4)	O5—Cl2—O7	112.3 (6)
C7—C6—C5	126.6 (4)	O6—Cl2—O7	108.2 (3)
C6—C7—C8	119.3 (4)	O8—Cl2—O7	107.4 (3)
C6—C7—H7	120.3	C39—C38—H38A	109.5
C8—C7—H7	120.3	C39—C38—H38B	109.5
C7—C8—C9	117.8 (4)	H38A—C38—H38B	109.5
C7—C8—C16	121.0 (4)	C39—C38—H38C	109.5
C9—C8—C16	121.2 (4)	H38A—C38—H38C	109.5
C10—C9—C8	120.5 (4)	H38B—C38—H38C	109.5
C10—C9—H9	119.8	N6—C39—C38	178.0 (8)
C8—C9—H9	119.8	O4—Cl1—O1'	119.7 (7)
N2—C10—C9	119.5 (4)	O4—Cl1—O4'	54.5 (7)
N2—C10—C11	113.5 (4)	O1'—Cl1—O4'	118.5 (7)
C9—C10—C11	127.0 (4)	O4—Cl1—O2	116.2 (7)
N3—C11—C12	121.3 (4)	O1'—Cl1—O2	116.4 (6)
N3—C11—C10	113.7 (4)	O4'—Cl1—O2	73.7 (7)
C12—C11—C10	125.0 (4)	O4—Cl1—O1	109.7 (7)
N5—C34—C33	123.3 (4)	O4'—Cl1—O1	93.0 (7)
N5—C34—C30	117.0 (4)	O2—Cl1—O1	107.5 (6)
C33—C34—C30	119.7 (4)	O4—Cl1—O3'	50.9 (6)
C11—C12—C13	119.3 (5)	O1'—Cl1—O3'	111.0 (6)
C11—C12—H12	120.4	O4'—Cl1—O3'	103.3 (7)
C13—C12—H12	120.4	O2—Cl1—O3'	127.1 (6)
C14—C13—C12	119.9 (5)	O1—Cl1—O3'	125.3 (6)
C14—C13—H13	120.1	O4—Cl1—O3	109.8 (7)
C12—C13—H13	120.1	O1'—Cl1—O3	86.0 (7)
C13—C14—C15	118.7 (5)	O4'—Cl1—O3	154.7 (7)
C13—C14—H14	120.6	O2—Cl1—O3	101.8 (6)
C15—C14—H14	120.6	O1—Cl1—O3	111.8 (7)
N3—C15—C14	121.5 (5)	O3'—Cl1—O3	59.0 (6)
N3—C15—H15	119.3	O4—Cl1—O2'	131.7 (7)
C14—C15—H15	119.3	O1'—Cl1—O2'	108.4 (6)
C20—C16—C17	107.0 (4)	O4'—Cl1—O2'	106.4 (7)
C20—C16—C8	127.0 (4)	O1—Cl1—O2'	115.8 (7)
C17—C16—C8	125.9 (4)	O3'—Cl1—O2'	108.8 (6)
C20—C16—Fe1	69.5 (3)	O3—Cl1—O2'	67.6 (7)
C17—C16—Fe1	69.5 (3)	Cl1—O1—Cu1	120.5 (6)
			
N2—Cu1—N1—C1	174.5 (4)	C22—Fe1—C19—C18	−54.4 (8)
N4—Cu1—N1—C1	−8.7 (4)	C17—Fe1—C19—C18	37.4 (3)
N3—Cu1—N1—C1	146.3 (4)	C21—Fe1—C19—C18	167.9 (8)
N5—Cu1—N1—C1	−88.3 (4)	C25—Fe1—C19—C20	74.7 (5)
O1—Cu1—N1—C1	76.1 (4)	C23—Fe1—C19—C20	156.8 (5)
N2—Cu1—N1—C5	−4.6 (3)	C24—Fe1—C19—C20	115.4 (5)
N4—Cu1—N1—C5	172.2 (3)	C16—Fe1—C19—C20	−37.5 (3)
N3—Cu1—N1—C5	−32.8 (5)	C22—Fe1—C19—C20	−173.9 (7)
N5—Cu1—N1—C5	92.6 (3)	C17—Fe1—C19—C20	−82.1 (3)
O1—Cu1—N1—C5	−103.0 (3)	C21—Fe1—C19—C20	48.5 (10)
N1—Cu1—N2—C10	−177.6 (3)	C18—Fe1—C19—C20	−119.5 (4)
N3—Cu1—N2—C10	−8.3 (3)	C17—C16—C20—C19	−0.5 (5)
N5—Cu1—N2—C10	89.5 (3)	C8—C16—C20—C19	−176.3 (4)
O1—Cu1—N2—C10	−90.7 (4)	Fe1—C16—C20—C19	−60.1 (3)
N1—Cu1—N2—C6	8.1 (3)	C17—C16—C20—Fe1	59.6 (3)
N3—Cu1—N2—C6	177.4 (3)	C8—C16—C20—Fe1	−116.2 (5)
N5—Cu1—N2—C6	−84.8 (3)	C18—C19—C20—C16	0.4 (6)
O1—Cu1—N2—C6	94.9 (4)	Fe1—C19—C20—C16	59.8 (3)
N2—Cu1—N3—C15	−174.3 (4)	C18—C19—C20—Fe1	−59.4 (4)
N4—Cu1—N3—C15	8.7 (4)	C25—Fe1—C20—C16	117.2 (5)
N1—Cu1—N3—C15	−146.1 (4)	C23—Fe1—C20—C16	−171.5 (6)
N5—Cu1—N3—C15	89.3 (4)	C24—Fe1—C20—C16	158.7 (4)
O1—Cu1—N3—C15	−75.1 (4)	C22—Fe1—C20—C16	52.4 (11)
N2—Cu1—N3—C11	5.5 (3)	C17—Fe1—C20—C16	−38.7 (3)
N4—Cu1—N3—C11	−171.4 (3)	C21—Fe1—C20—C16	78.6 (5)
N1—Cu1—N3—C11	33.8 (6)	C18—Fe1—C20—C16	−82.4 (3)
N5—Cu1—N3—C11	−90.8 (3)	C19—Fe1—C20—C16	−119.4 (4)
O1—Cu1—N3—C11	104.8 (3)	C25—Fe1—C20—C19	−123.3 (5)
N1—Cu1—N4—C26	88.2 (4)	C23—Fe1—C20—C19	−52.0 (8)
N3—Cu1—N4—C26	−82.1 (4)	C24—Fe1—C20—C19	−81.9 (5)
N5—Cu1—N4—C26	−179.4 (4)	C16—Fe1—C20—C19	119.4 (4)
O1—Cu1—N4—C26	0.9 (5)	C22—Fe1—C20—C19	171.8 (9)
N1—Cu1—N4—C30	−88.0 (3)	C17—Fe1—C20—C19	80.8 (3)
N3—Cu1—N4—C30	101.7 (3)	C21—Fe1—C20—C19	−162.0 (5)
N5—Cu1—N4—C30	4.4 (3)	C18—Fe1—C20—C19	37.0 (3)
O1—Cu1—N4—C30	−175.4 (4)	C20—Fe1—C21—C25	72.0 (7)
N2—Cu1—N5—C37	0.3 (5)	C23—Fe1—C21—C25	−82.1 (6)
N4—Cu1—N5—C37	−179.5 (5)	C24—Fe1—C21—C25	−38.2 (6)
N1—Cu1—N5—C37	−80.4 (5)	C16—Fe1—C21—C25	114.3 (6)
N3—Cu1—N5—C37	80.9 (5)	C22—Fe1—C21—C25	−119.8 (9)
O1—Cu1—N5—C37	−178.7 (10)	C17—Fe1—C21—C25	157.6 (5)
N2—Cu1—N5—C34	174.5 (3)	C18—Fe1—C21—C25	−166.6 (7)
N4—Cu1—N5—C34	−5.3 (3)	C19—Fe1—C21—C25	35.7 (12)
N1—Cu1—N5—C34	93.8 (3)	C25—Fe1—C21—C22	119.8 (9)
N3—Cu1—N5—C34	−104.9 (3)	C20—Fe1—C21—C22	−168.2 (5)
O1—Cu1—N5—C34	−4.5 (11)	C23—Fe1—C21—C22	37.8 (6)
C5—N1—C1—C2	−1.2 (7)	C24—Fe1—C21—C22	81.6 (6)
Cu1—N1—C1—C2	179.8 (4)	C16—Fe1—C21—C22	−125.9 (6)
N1—C1—C2—C3	0.7 (8)	C17—Fe1—C21—C22	−82.6 (6)
C1—C2—C3—C4	−0.7 (7)	C18—Fe1—C21—C22	−46.8 (11)
C2—C3—C4—C5	1.1 (7)	C19—Fe1—C21—C22	155.5 (8)
C1—N1—C5—C4	1.6 (6)	C25—C21—C22—C23	−1.3 (9)
Cu1—N1—C5—C4	−179.2 (3)	Fe1—C21—C22—C23	−60.3 (5)
C1—N1—C5—C6	−178.2 (4)	C25—C21—C22—Fe1	59.0 (6)
Cu1—N1—C5—C6	0.9 (4)	C25—Fe1—C22—C23	81.7 (6)
C3—C4—C5—N1	−1.6 (7)	C20—Fe1—C22—C23	152.3 (9)
C3—C4—C5—C6	178.2 (4)	C24—Fe1—C22—C23	37.9 (5)
C10—N2—C6—C7	−1.7 (6)	C16—Fe1—C22—C23	−167.3 (5)
Cu1—N2—C6—C7	172.4 (3)	C17—Fe1—C22—C23	−123.5 (6)
C10—N2—C6—C5	176.1 (4)	C21—Fe1—C22—C23	118.1 (8)
Cu1—N2—C6—C5	−9.7 (5)	C18—Fe1—C22—C23	−79.9 (6)
N1—C5—C6—N2	5.4 (5)	C19—Fe1—C22—C23	−41.1 (11)
C4—C5—C6—N2	−174.5 (4)	C25—Fe1—C22—C21	−36.4 (5)
N1—C5—C6—C7	−176.9 (4)	C20—Fe1—C22—C21	34.2 (13)
C4—C5—C6—C7	3.2 (7)	C23—Fe1—C22—C21	−118.1 (8)
N2—C6—C7—C8	−0.6 (6)	C24—Fe1—C22—C21	−80.2 (6)
C5—C6—C7—C8	−178.1 (4)	C16—Fe1—C22—C21	74.5 (7)
C6—C7—C8—C9	2.0 (6)	C17—Fe1—C22—C21	118.4 (6)
C6—C7—C8—C16	−178.9 (4)	C18—Fe1—C22—C21	162.0 (5)
C7—C8—C9—C10	−1.4 (7)	C19—Fe1—C22—C21	−159.2 (7)
C16—C8—C9—C10	179.5 (4)	C21—C22—C23—C24	0.4 (9)
C6—N2—C10—C9	2.4 (6)	Fe1—C22—C23—C24	−60.1 (5)
Cu1—N2—C10—C9	−171.7 (3)	C21—C22—C23—Fe1	60.4 (6)
C6—N2—C10—C11	−176.7 (4)	C25—Fe1—C23—C22	−80.9 (6)
Cu1—N2—C10—C11	9.3 (5)	C20—Fe1—C23—C22	−161.0 (7)
C8—C9—C10—N2	−0.8 (6)	C24—Fe1—C23—C22	−118.6 (8)
C8—C9—C10—C11	178.1 (4)	C16—Fe1—C23—C22	39.8 (13)
C15—N3—C11—C12	−0.6 (6)	C17—Fe1—C23—C22	77.9 (7)
Cu1—N3—C11—C12	179.5 (3)	C21—Fe1—C23—C22	−38.4 (6)
C15—N3—C11—C10	177.5 (4)	C18—Fe1—C23—C22	119.8 (6)
Cu1—N3—C11—C10	−2.4 (5)	C19—Fe1—C23—C22	162.2 (6)
N2—C10—C11—N3	−4.0 (5)	C25—Fe1—C23—C24	37.7 (5)
C9—C10—C11—N3	177.0 (4)	C20—Fe1—C23—C24	−42.4 (9)
N2—C10—C11—C12	174.0 (4)	C16—Fe1—C23—C24	158.4 (9)
C9—C10—C11—C12	−5.0 (7)	C22—Fe1—C23—C24	118.6 (8)
C37—N5—C34—C33	1.4 (7)	C17—Fe1—C23—C24	−163.5 (5)
Cu1—N5—C34—C33	−173.8 (4)	C21—Fe1—C23—C24	80.2 (6)
C37—N5—C34—C30	−179.5 (4)	C18—Fe1—C23—C24	−121.6 (6)
Cu1—N5—C34—C30	5.3 (5)	C19—Fe1—C23—C24	−79.2 (6)
N3—C11—C12—C13	0.9 (7)	C22—C23—C24—C25	0.6 (9)
C10—C11—C12—C13	−176.9 (4)	Fe1—C23—C24—C25	−59.6 (5)
C11—C12—C13—C14	−0.4 (8)	C22—C23—C24—Fe1	60.2 (5)
C12—C13—C14—C15	−0.4 (8)	C20—Fe1—C24—C25	−80.4 (6)
C11—N3—C15—C14	−0.3 (7)	C23—Fe1—C24—C25	118.9 (8)
Cu1—N3—C15—C14	179.6 (4)	C16—Fe1—C24—C25	−47.9 (9)
C13—C14—C15—N3	0.8 (8)	C22—Fe1—C24—C25	81.3 (6)
C7—C8—C16—C20	−10.9 (7)	C17—Fe1—C24—C25	168.2 (8)
C9—C8—C16—C20	168.1 (5)	C21—Fe1—C24—C25	37.2 (6)
C7—C8—C16—C17	174.0 (5)	C18—Fe1—C24—C25	−162.5 (6)
C9—C8—C16—C17	−7.0 (7)	C19—Fe1—C24—C25	−122.8 (6)
C7—C8—C16—Fe1	−98.8 (5)	C25—Fe1—C24—C23	−118.9 (8)
C9—C8—C16—Fe1	80.2 (5)	C20—Fe1—C24—C23	160.7 (5)
C25—Fe1—C16—C20	−79.8 (5)	C16—Fe1—C24—C23	−166.8 (6)
C23—Fe1—C16—C20	166.5 (10)	C22—Fe1—C24—C23	−37.6 (5)
C24—Fe1—C16—C20	−46.3 (7)	C17—Fe1—C24—C23	49.2 (11)
C22—Fe1—C16—C20	−162.6 (5)	C21—Fe1—C24—C23	−81.7 (6)
C17—Fe1—C16—C20	118.2 (4)	C18—Fe1—C24—C23	78.6 (6)
C21—Fe1—C16—C20	−121.2 (5)	C19—Fe1—C24—C23	118.3 (6)
C18—Fe1—C16—C20	80.6 (3)	C22—C21—C25—C24	1.7 (9)
C19—Fe1—C16—C20	37.7 (3)	Fe1—C21—C25—C24	60.6 (5)
C25—Fe1—C16—C17	162.0 (5)	C22—C21—C25—Fe1	−58.8 (6)
C20—Fe1—C16—C17	−118.2 (4)	C23—C24—C25—C21	−1.5 (9)
C23—Fe1—C16—C17	48.2 (11)	Fe1—C24—C25—C21	−61.2 (5)
C24—Fe1—C16—C17	−164.6 (6)	C23—C24—C25—Fe1	59.7 (5)
C22—Fe1—C16—C17	79.2 (5)	C20—Fe1—C25—C21	−125.2 (7)
C21—Fe1—C16—C17	120.5 (5)	C23—Fe1—C25—C21	80.8 (6)
C18—Fe1—C16—C17	−37.7 (3)	C24—Fe1—C25—C21	118.5 (9)
C19—Fe1—C16—C17	−80.6 (3)	C16—Fe1—C25—C21	−83.5 (7)
C25—Fe1—C16—C8	41.8 (6)	C22—Fe1—C25—C21	37.6 (6)
C20—Fe1—C16—C8	121.6 (5)	C17—Fe1—C25—C21	−52.6 (11)
C23—Fe1—C16—C8	−72.0 (11)	C18—Fe1—C25—C21	165.9 (8)
C24—Fe1—C16—C8	75.2 (8)	C19—Fe1—C25—C21	−166.2 (6)
C22—Fe1—C16—C8	−41.0 (6)	C20—Fe1—C25—C24	116.3 (6)
C17—Fe1—C16—C8	−120.2 (5)	C23—Fe1—C25—C24	−37.6 (5)
C21—Fe1—C16—C8	0.3 (6)	C16—Fe1—C25—C24	158.0 (5)
C18—Fe1—C16—C8	−157.9 (5)	C22—Fe1—C25—C24	−80.9 (6)
C19—Fe1—C16—C8	159.2 (4)	C17—Fe1—C25—C24	−171.1 (6)
C20—C16—C17—C18	0.4 (6)	C21—Fe1—C25—C24	−118.5 (9)
C8—C16—C17—C18	176.2 (4)	C18—Fe1—C25—C24	47.4 (11)
Fe1—C16—C17—C18	60.0 (4)	C19—Fe1—C25—C24	75.3 (6)
C20—C16—C17—Fe1	−59.6 (3)	C30—N4—C26—C27	−1.2 (7)
C8—C16—C17—Fe1	116.3 (5)	Cu1—N4—C26—C27	−177.3 (4)
C25—Fe1—C17—C18	−161.3 (8)	N4—C26—C27—C28	1.0 (8)
C20—Fe1—C17—C18	−80.8 (3)	C26—C27—C28—C29	−1.1 (8)
C23—Fe1—C17—C18	76.0 (5)	C27—C28—C29—C30	1.3 (8)
C24—Fe1—C17—C18	38.2 (11)	C27—C28—C29—C31	179.4 (5)
C16—Fe1—C17—C18	−118.8 (5)	C26—N4—C30—C29	1.5 (7)
C22—Fe1—C17—C18	118.0 (6)	Cu1—N4—C30—C29	177.9 (3)
C21—Fe1—C17—C18	161.5 (6)	C26—N4—C30—C34	−179.5 (4)
C19—Fe1—C17—C18	−36.7 (3)	Cu1—N4—C30—C34	−3.1 (5)
C25—Fe1—C17—C16	−42.5 (9)	C28—C29—C30—N4	−1.5 (7)
C20—Fe1—C17—C16	38.0 (3)	C31—C29—C30—N4	−179.7 (5)
C23—Fe1—C17—C16	−165.2 (5)	C28—C29—C30—C34	179.5 (4)
C24—Fe1—C17—C16	157.0 (9)	C31—C29—C30—C34	1.2 (7)
C22—Fe1—C17—C16	−123.2 (5)	N5—C34—C30—N4	−2.1 (6)
C21—Fe1—C17—C16	−79.7 (6)	C33—C34—C30—N4	177.0 (4)
C18—Fe1—C17—C16	118.8 (5)	N5—C34—C30—C29	177.0 (4)
C19—Fe1—C17—C16	82.1 (3)	C33—C34—C30—C29	−3.9 (7)
C16—C17—C18—C19	−0.1 (6)	C28—C29—C31—C32	−176.5 (5)
Fe1—C17—C18—C19	59.3 (4)	C30—C29—C31—C32	1.6 (8)
C16—C17—C18—Fe1	−59.4 (3)	C29—C31—C32—C33	−1.7 (9)
C25—Fe1—C18—C19	37.6 (10)	N5—C34—C33—C32	−177.1 (4)
C20—Fe1—C18—C19	−37.8 (3)	C30—C34—C33—C32	3.9 (7)
C23—Fe1—C18—C19	115.1 (5)	N5—C34—C33—C35	0.2 (7)
C24—Fe1—C18—C19	73.0 (5)	C30—C34—C33—C35	−178.9 (4)
C16—Fe1—C18—C19	−81.6 (3)	C31—C32—C33—C34	−1.0 (8)
C22—Fe1—C18—C19	157.0 (5)	C31—C32—C33—C35	−178.1 (6)
C17—Fe1—C18—C19	−120.0 (4)	C34—C33—C35—C36	−0.2 (8)
C21—Fe1—C18—C19	−168.3 (9)	C32—C33—C35—C36	177.0 (5)
C25—Fe1—C18—C17	157.6 (8)	C33—C35—C36—C37	−1.3 (9)
C20—Fe1—C18—C17	82.2 (3)	C34—N5—C37—C36	−3.0 (8)
C23—Fe1—C18—C17	−124.9 (5)	Cu1—N5—C37—C36	170.7 (4)
C24—Fe1—C18—C17	−167.0 (4)	C35—C36—C37—N5	3.1 (9)
C16—Fe1—C18—C17	38.4 (3)	O4—Cl1—O1—Cu1	−177.0 (8)
C22—Fe1—C18—C17	−83.0 (5)	O1'—Cl1—O1—Cu1	−58.9 (15)
C21—Fe1—C18—C17	−48.3 (10)	O4'—Cl1—O1—Cu1	129.8 (8)
C19—Fe1—C18—C17	120.0 (4)	O2—Cl1—O1—Cu1	55.9 (9)
C17—C18—C19—C20	−0.2 (6)	O3'—Cl1—O1—Cu1	−121.6 (7)
Fe1—C18—C19—C20	58.6 (3)	O3—Cl1—O1—Cu1	−54.9 (9)
C17—C18—C19—Fe1	−58.7 (4)	O2'—Cl1—O1—Cu1	19.9 (11)
C25—Fe1—C19—C18	−165.8 (4)	N2—Cu1—O1—Cl1	14.4 (8)
C20—Fe1—C19—C18	119.5 (4)	N4—Cu1—O1—Cl1	−165.9 (7)
C23—Fe1—C19—C18	−83.7 (5)	N1—Cu1—O1—Cl1	94.2 (7)
C24—Fe1—C19—C18	−125.1 (5)	N3—Cu1—O1—Cl1	−64.2 (7)
C16—Fe1—C19—C18	82.0 (3)	N5—Cu1—O1—Cl1	−166.6 (4)
